# Why Most Research Findings About Psi Are False: The Replicability Crisis, the Psi Paradox and the Myth of Sisyphus

**DOI:** 10.3389/fpsyg.2020.562992

**Published:** 2020-09-18

**Authors:** Thomas Rabeyron

**Affiliations:** ^1^Université de Lorraine, Interpsy, Nancy, France; ^2^University of Edinburgh, KPU, Edinburgh, United Kingdom

**Keywords:** psi, precognition, replicability crisis, pre-registration, methodology

## Abstract

The replicability crisis in psychology has been influenced by the results of nine experiments conducted by Bem (2011) and presented as supporting the existence of precognition. In this paper, we hope to show how the debate concerning these experiments could be an opportunity to develop original thinking about psychology and replicability. After a few preliminary remarks about psi and scientific epistemology, we examine how psi results lead to a paradox which questions how appropriate the scientific method is to psi research. This paradox highlights a problem in the way experiments are conducted in psi research and its potential consequence on mainstream research in psychology. Two classical experiments – the Ganzfeld protocol and the Bem studies – are then analyzed in order to illustrate this paradox and its consequences. Mainstream research is also addressed in the broader context of the replication crisis, decline effect and questionable research practices. Several perspectives for future research are proposed in conclusion and underline the heuristic value of psi studies for psychology.

## Replicability Crisis and Psi Research

The replicability crisis has been illustrated by the results of nine experiments conducted by Bem (2011) and reported in the *Journal of Personality and Social Psychology* as supporting the existence of precognition. As Romero (2019) explains “although the finding persuaded very few scientists, the controversy engendered mistrust in the ways psychologists conduct their experiments because Bem used procedures and statistical tools that many social psychologists use” (p. 3). Indeed, if Bem was able to demonstrate the existence of precognition – and given that precognition cannot exist for a lot of psychologists (Reber and Alcock, 2020) – did he show unwittingly that something was profoundly wrong in the way experiments are conducted in the field of psychology (Wiggins and Chrisopherson, 2019)? Many relevant papers have been published since Bem’s initial publication (Pashler and Harris, 2012; Savalei and Dunn, 2015) about the replicability crisis, Bayesian statistics (Witte and Zenker, 2017), and questionable research practices (QRPs; Wagenmakers et al., 2011; Bierman et al., 2016). In the present paper, we would like to suggest that this debate could be an opportunity to develop original thinking about psychology and replicability. In this regard, we will show that the Bem studies are not an isolated “accident,” but are actually inserted in a long tradition of research which tries to deal with complex epistemological problems concerning the nature of reality and human consciousness. Specifically, we will argue that the controversies about the existence of psi could be highly informative about psychology and consciousness studies.

Psi research can be considered as a subfield of consciousness studies concerned with interactions between individuals and their environment that transcends the ordinary constraints of space-time (Bem and Honorton, 1994; Radin, 2006; Irwin and Watt, 2007; Cardeña, 2018). Different lines of research have been developed for more than a century to tackle psi using experimental research (Rhine et al., 1966), spontaneous cases (Rhine-Feather and Schmicker, 2005), clinical cases (Rabeyron, 2020), selected participants^1^ (Méheust, 1999; Braude, 2007), and applications (Schwartz, 2001, 2007; May et al., 2018). Several meta-analyses of studies conducted under controlled conditions examine precognitive dreams (*es* = 0.14; Sherwood and Roe, 2003), telepathy (*es* = 0.14; Storm et al., 2010), and presentiment (*es* = 0.21; Mossbridge et al., 2012) and have demonstrated statistically significant effects. Bem (2011) research about precognition^2^ published in the JPSP is thus not isolated. It can be considered as the logical of evolution of previous psi research.

While these results support the existence of consistent anomalous experience/behavior that has been labeled “psi,” there is currently no consensus in the scientific community concerning their interpretation and two main positions have emerged so far. The “skeptics” suppose that they are the consequences of errors, bias, and different forms of QRPs (Alcock, 2003; Alcock et al., 2003; Hyman, 2010; Wiseman, 2010; Wagenmakers et al., 2011; Reber and Alcock, 2020). The “proponents” argue that these results prove the existence of psi beyond reasonable doubt and that new research should move on to the analysis of psi processes rather than yet more attempts to prove its existence (Radin, 2006; Cardeña et al., 2015; Cardeña, 2018). This absence of consensus is related to the difficulty of drawing firm conclusions from the results of psi research. Indeed, they represent an anomaly (Rao and Palmer, 1987) because there is currently no scientific model – based on physical or biology principles – to explain such interactions even if they exist (Kuhn, 1962)^3^. Nevertheless, which ever explanation is correct, the results of psi research may be informative for the wider psychological sciences (Schooler et al., 2018). Indeed, they lead to two opposites but very heuristic hypothesis: (a) within this domain of research, which has been conducted by hundreds of researchers whose critical efforts span over a century, the researchers have either been fraudulent or have been fooled, even when using the most reliable tools of scientific research and (b) psi exists and human consciousness can interact with its environment beyond the usual boundaries of space and time. This paper will explore these two hypotheses and their consequences for psi research and psychology in general.

## What if… Psi *Really* Exists? The Psi Paradox

History of science has shown many examples of phenomena that were observed in a reliable manner but which were rejected by the scientific community because they were not explainable at the time of their observation (e.g., meteorites, heartbeat, etc.). Consequently, it might be wise to be careful when considering anomalous results of the psi variety, especially knowing that 22 Nobel Prize winners, leading scientists, and figures of the intellectual life have reported such experiences and took a position in favor of their existence (Méheust, 1999)^4^. Thus, if we suppose – at least for a moment – that there are enough elements to take *seriously* the hypothesis that the (b) option is true, that is, psi *really* exists, what are the consequences of such an assumption? Could it make sense of other observations in the field of psychology and other scientific domains? And does it change the way scientific research should be conducted?

The psi studies, viewed as a whole, suggest that a kind of “direct” interaction (conscious or unconscious) between individual humans and their environment is possible. This interaction concerns events or objects situated at a distance in space and time (Mossbridge and Radin, 2018). It can take many forms (gut feeling, behavior, mental representation, etc.) with different intensities (e.g., a small or a strong emotion). It can be perceptive (from the environment to the person) or projective (from the person to the environment). It can be associated with a transfer of information or, rather, something that *looks like* a transfer of information (Lucadou et al., 2007). It emerges more easily during altered states of consciousness (Storm et al., 2010), is more pronounced for some persons (Schlitz and Honorton, 1992), and tends to emerge during or after traumatic events (Rabeyron and Loose, 2015). This direct interaction is more generally associated with subjective paranormal experiences (e.g., near death experiences and out of body experiences) even if they do not overlap entirely (Rabeyron et al., 2018). Moreover, even if the role of consciousness in psi processes is not well understood, it is likely that attention (McMoneagle and May, 2014), memory (Carpenter, 2012), creativity (Holt, 2012), personality traits (Thalbourne, 2000), belief (Lawrence, 1993), and psychodynamic aspects (Rabeyron and Loose, 2015) are components of it.

These results from psi research construct a specific paradox that is the crux of the present paper. This paradox would lead to the conclusion that most research findings in psi research are *false* (or inappropriate) but not for the reasons usually supposed by the skeptics. We need first to recall that scientific research is based on the principle that, in the main, the researcher (the observer) is separated from, or independent of, the dependent variable. The researcher tries not to affect the result of an experiment so that what is observed varies with the independent variable being tested and is independent of the researcher’s thoughts, intentions, belief, or disbelief. This assumption is assumed to permit other scientists to demonstrate the same result under the same conditions (or not, as the case may be). This is the logical and epistemological frame in which scientific research is usually conducted. This model works very well and has produced much reliable scientific knowledge and technological progress. Even if this is a simplistic vision of the way scientific research progresses, as shown by epistemology of science (Feyerabend, 1975; Chalmers, 1979), such a representation of scientific research is nevertheless a useful principle, which guides scientists to good practice.

However, if a direct interaction (in the sense described above) between a person and their environment is possible, this principle too could influence the outcome of experiments purporting to use the scientific principles, because there *could* be a direct interaction between the scientists (the observer) and their object of study (the observed). Thus, if psi exists, the problem is the following: *an advertent or inadvertent “direct” interaction between the researcher and the object of study could be possible. This destroys the conditions necessary for the convincing scientific demonstration of psi itself*.

This leads to the following paradoxs: (1) if the existence of psi is proven in a classical scientific setting, then it demonstrates retroactively that this setting is inappropriate because psi implies that there is no clear “cut” between the observer and the observed. (2) So, if this assumption of the scientific setting is inappropriate (because of psi), then this setting cannot be used to prove the existence of psi. But, then, as psi cannot be proven, the scientific setting itself appears as being still appropriate! Consequently, we can try to use the scientific setting to prove the existence of psi but then we are now again at (1), which leads to (2), and it logically follows an infinite paradoxical loop between (1) and (2). It shows more generally that the principle on which a scientific experiment is based to study psi – the ontological separation between the observer and the observed – would be erroneous. Consequently, any scientific knowledge in this field of research could not be produced as soon as there is no clear distinction between the observer and the observed. Indeed, there is no way to know, for epistemological reasons, if what is observed is an effect induced by the experimenter (due to a possible psi influence) or a characteristic of the phenomena independently of the experimenter. The distinction between expectations and reality is then unclear, and the psi researcher using this approach can only become a modern version of Sisyphus^5^ as it will be shown later.

## Two Examples From the Literature: the Ganzfeld Protocol and the Bem Studies

The Ganzfeld experiment – the most classical protocol in psi research (Bem and Honorton, 1994) – provides an interesting example of the previous reasoning. During this experiment, a participant (the receiver) is comfortably seated in a chair, wearing a mask showing him a uniform visual field (usually red) while listening to a white noise. The participant is immersed in a constant and neutral sensory field that rapidly engenders an altered state of consciousness supposed to favor psi perceptions (Storm et al., 2010). After 20 min, the participant tries to find, among several images or videos (usually, the correct target and three alternate targets), the one that was “transmitted” telepathically by another participant (the sender) located in another room. As shown by the literature, an average success rate of 33% in the receiver’s choice has been obtained instead of 25% by chance alone (Storm et al., 2010). A significant correlation between the choices of the receivers and the targets shown to the senders has thus been demonstrated. But what else has been learned and does this experiment demonstrate the existence of telepathy?

No, because it cannot be proven in a definite manner. Different competing interpretations could be proposed but they cannot be isolated or confirmed. There is indeed no way to propose a *falsifiable claim* or set-up a crucial experiment (Popper, 1934). For example, is it a telepathic effect (the target is “sent” by the sender and “received” by the receiver) or a precognitive effect (the receiver actually perceives the target from the “future,” when presented with the four possible targets at the end of the session and asked to choose)? To test this hypothesis, an experiment can be set-up in which a feedback is given to half of the receivers (they see the correct target after the Ganzfeld state) and no feedback is given to half of the receivers (they do not see the correct target and, instead, a blind judge evaluates the correlation between the receiver’s mentations and the four possible targets)^6^. If we suppose that a significant effect is obtained only for feedback trials, does it prove the precognitive hypothesis? Not so, because even if this experiment was replicated 50 times with the same results and using the best experimental conditions, there is no way to know if this effect is a consequence of (1) a precognitive effect, (2) a psi influence from the participants, (3) a psi effect from the experimenter on the computer that chooses the target, and (4) many other options! Because as soon as there is no clear *cut* between the experimenter, the participant, and the methodology, everything becomes possible.

Another possibility is that some experimenters are consistently able to influence the experimental data and so gain significant results. This variable itself has been the focus of considerable research (Broughton, 1979; Palmer, 1997; Parker and Miller, 2014)^7^. It can be tested during a Ganzfeld experiment by working with 10 different experimenters and by comparing their results. But even if a correlation between (for example) the experimenter’s belief (a measure of its possible psi influence on the setup) and the Ganzfeld effect size is found, no clear conclusion could be reached. It could be (1) the effect of the experimenter, (2) the effect of the analyst of the study (the one who look at the data first), or (3) many other potential explanations! This is again the same problem: if there is no clear cut between the experimenter, the participants, and the scientific set-up, there is no falsifiable claim. Importantly, *this problem is infinite*: when new variables are introduced, without a clearly falsifiable hypothesis (due to the absence of an epistemological boundary between the observer and what is observed), the problem exists. This lack of clear attributable causation is relevant to the results of most psi experiments and that’s *why the psi effects are probably not what they look like*. They can be considered as inconclusive from a scientific point of view. Thus, there is no way to be sure that the Ganzfeld is a transfer of information between two people or that precognition is the ability to extract information from the future. Psi studies actually just show that significant correlations between two variables – an intention and a measure – will emerge and take different forms depending on the conditions of the experimental setting.

The recent experiments reported by Bem (2011) on the anomalous anticipation of random events illustrate perfectly the problem we have just described. Bem (2011), publishing in a mainstream psychological journal, the *Journal of Personality and Social Psychology*, presented the results of four classical psychology experiments (e.g., priming task) that he has “reversed” in order to see if the participants would be influenced by stimuli not from the past but from the *future*. This publication induced considerable controversy between skeptics and proponents in the academic community and even in several mainstream media (Bem et al., 2011; Wagenmakers et al., 2011; Ritchie et al., 2012). We can sum up the different steps of this research paradigm: (1) Bem (2011) shows a seemingly reliable psi effect in controlled condition. It engenders critical reactions from the mainstream community (Wagenmakers et al., 2011). In a way, these critics are right: they probably “feel” that it does not look like a classical effect and maybe understand intuitively the potential consequences of this result for the whole field of psychology. (2) The experiment is then replicated in different settings (online, different populations, different stimuli, etc.)^8^. Sometimes it works, sometimes it does not work (Galak et al., 2012; Ritchie et al., 2012). (3) A meta-analysis is finally published with a significant, but smaller effect size (*es* = 0.14), than the original publication (*es* = 0.20; Bem et al., 2015)^9^. Is it because: (a) the effect actually does not exist; (b) the setting of the new experiments was less appropriate; (c) the replication process in itself decreases the effect? (Lucadou, 1995; Kennedy, 2003); or (d) the effect is actually the consequence of an experimenter effect described above? (Kennedy and Taddonio, 1976; Broughton, 1979; Palmer, 1997; Smith, 2003; Parker and Miller, 2014). A fourth step is thus necessary; and (4) a new study is concurrently conducted in different labs to study the impact of experimenter’s individual differences on the results. But this effect could actually depend on the analyst (i.e., the one who look at the results for the first time or analyze the data) and not the experimenter (West and Fisk, 1953). So, the next step of this research paradigm might be (5) to test the magnitude of the effect with 10 different experimenters and 10 different analysts.

These different steps show the possible impediments which plague psi researchers in their efforts to prove the existence of psi, in addition to negative results and criticism from mainstream colleagues. And even if the researcher manages to get significant results at every step – as done by Bem (2011) for many years^10^ –, there will always be new demands from the mainstream community: more control of the experimental setting, more experiments, more labs, more statistical tools, etc., (Wagenmakers et al., 2015)^11^. Even when proponents manage to agree a clear protocol with skeptics, and then obtain significant results, which has been the case with the Ganzfeld (Hyman and Honorton, 1986; Bem and Honorton, 1994), it is never enough. The underlying problem is that even if a significant effect is found at each step, there is no way to conclude anything about the nature of the effect and consequently no way to produce scientific knowledge about the *source* of psi (Broughton, 1979; Palmer, 1997): is it from the participants? From the experimenter? Is it from each experimenter separately? Or is it a stronger influence from the first one who analyze the data? Or, maybe that the one who has originally conceived the experiment? Are there degrees of influence between the experimenter and the participants depending on the type of experiment? But also, is it an effect in the present (during the experiment) or is it an influence from the future (after the experiment), or even the past, if psi can transcend space and time? There are no definite answers to these questions, whatever results are obtained and unfortunately, there is, to our knowledge, no way to answer these questions because there are only *plausible interpretations*. Fundamentally, the problem is that the usual epistemological frame of research is not adequate when considering psi proprieties.

In this regard, it might be relevant to stop doing research whose aim is to prove the existence of psi using classical (scientific method) setting, because it does not really make sense from an epistemological point of view. It may be argued that this methodology cannot produce anything new even with large financing and the passage of a century of research. Nevertheless, these experiments are relevant in terms of *ritual*. A selection of classical psi experiments can be used – e.g., the Ganzfeld, dreams or presentiment studies – as illustrations of a phenomena, recognizing their “limitations” and without believing that something new explanation will be emerge from them. If they are conducted with enough intention, motivation, novelty, and creativity, these experiments should continue to produce significant results. Their interest is mainly strategic because it gives the opportunity to show that psi can be replicated and produce significant effects in controlled conditions as shown by several meta-analysis (Cardeña, 2018).

Nevertheless, these “demonstration studies” might even be more complicated if we also take into account that psi effects tend to disappear when the same experiment is replicated, which is described as the *elusive* nature of psi (Hansen, 2001; Kennedy, 2003). When a psi experiment is set-up, a distinction between two variables or a hypothesis is proposed (true/false). If another experiment uses the same hypothesis, many researchers have reported that the effect tends to disappear (Kennedy, 2003)^12^. In this regard, using the same hypothesis twice for a psi experiment could be like asking a comedian trying to make the public laugh with the same joke twice. Psi interactions seem to be the expression of a novelty and novelty, by definition, can be new only once. It might explain the strange results – inversion, displacement, and disappearance of the effect – that appear when the same experiment is replicated (Lucadou, 1995). In order to avoid this difficulty, and following the Sisyphus metaphor, some researchers take a small rock (an experiment), push it up the mountain, and then do this with another rock (another experiment), but they do not push the same rock twice to avoid too much “resistance” that would result from the replication of the same experiment. They do not do this because the effect does not exist, as suggested by Wiseman (2010), but as the consequence of the fact that this is the only way to maintain the effect.

Due to these different epistemological difficulties, new knowledge in the field of psi research based on “classical” protocol would not be reliable and even those related to the understanding of psi processes. For example, if personality traits are correlated with psi, how to be sure this is not the consequence of the psi influence from the experimenter about which personality traits he believes favor psi? This is the same for all parameters that could be correlated with psi results^13^. These researches cannot produce scientific knowledge and so may be considered as a waste of time and energy in the same way as Sisyphus spends all his time doing a useless and infinite task. Some researchers in the field have recognized this problem and have stopped doing this kind of experimental research (Eisenbud, 1966, 1983). Others understand this problem and try to find a way to avoid it with specific experimental set-up (Lucadou et al., 2007; Walach and von Stillfried, 2011). Others acknowledge this problem but continue to do experimental research like this because this approach is relevant from a “political” and strategic point of view (Radin, 2018). They know intuitively how to conduct experiments in order to keep “alive” a psi effect in spite of its profoundly elusive nature. They suppose that these experiments can be useful to convince the whole scientific community, and a larger audience, if they are conducted in a sufficient rigorous way. This could be considered as a pragmatic approach using the wrong tools to show something that might be true. And other researchers do not understand this problem and continue to do this kind of research, in the same manner as Rhine (1966) used to do, because they do not feel that there are other options. It would be like a woodsman trying to fell trees with a feather saying that he continues to do so because this is the only tool he has. If they are lucky, despite the inappropriate nature of the research methodology, they will occasionally obtain significant results, but will also obtain null results. If they are resilient, they will do this during all their careers and become Sisyphus, trying to convince a scientific community who do not believe in the existence of what they study. Not surprisingly, some of them will stop doing parapsychological research and even can become skeptic (Blackmore, 1987).

## Replicability Crisis, Decline Effect, and Psi

As mentioned in the introduction, psychology and medicine have been confronted for more than 10 years by what has been called the replication or replicability crisis (Maxwell et al., 2015). It shows that the magnitude of the effect sizes in replications of psychology experiments is half the size of the original studies and that only 36% of the effect may be replicable (Nosek et al., 2015). The same problem is also true for other domains, especially medical and psychotherapy research (Ioannidis, 2016a,b). This is, of course, a huge problem that many researchers are trying to solve. One of the hypotheses to explain this situation is that these results could be the consequence of QRPs (John et al., 2012) and that most of these studies would not have been significant if they had been carried out with more rigor (Simmons et al., 2011)^14^. Consequently, the conduct of “Science” has to change by improving using – notably – pre-registration, better statistics, and the publication of null results.

The replication crisis has also underlined a phenomenon calls the “decline effect” (Schooler, 2011; Simmons et al., 2011; Protzko and Schooler, 2017). It shows that different effects in psychology and medicine tend to diminish with time and replication process (e.g., see Coyne and de Voogd, 2012; Gong and Jiao, 2019). In this regard, it is interesting to note that the psi community has reported such a decline effect a long time ago (Kennedy, 2003). Is it the same effect and what is its nature? Most researchers suppose that it is also the consequence of QRP^15^ but a different hypothesis could be proposed; the underlying problem of this decline effect might be psi, if the latter exists. It means that a direct relationship between an intention and reality is possible. Consequently, when mainstream research is set-up, psi might come in the equation even if it is not invited to the party^16^. When researchers develop a new protocol or hypothesis, their expectations or intentions might, through psi, unconsciously induce a result which favors their view. Thus, when a new effect or a new treatment is tested – with, for example, a control group – the researcher might have a psi influence at various points in the research design^17^, which could compromise the utility of the control group as a comparison condition.

As an example of how this unexpected influence could might affect data, May (1995) has developed the decision augmentation theory (DAT) to explain the results of studies in which participants had to influence, solely by intention, the output of a random number generator (Bösch et al., 2006). May supposes that there is no physical influence in this process and that the participant, using precognitive abilities, will choose (unconsciously) the right moment to push the button in order to get desired significant result^18^. In the same way, a researcher might unconsciously choose the right moment to start the study, choose the participants, collect the data, etc., in order to induce an effect in randomness. From this point of view, psi does not induce a transfer of energy or rely on a known physical force. It rather organizes reality in a discreet manner by ordering randomness. Consequently, some of the mainstream effects look like normal effects but they are not. It is only when other researchers – who may not have the same expectations, beliefs, or intentions – try to replicate them that these effects may mysteriously vanish. This would not be the effect of QRP, but the consequence of psi^19^.

It could be argued that if this hypothesis is true, there is no possibility of accumulating *any* reliable scientific knowledge. But this is not the case because all observed effects are not attributable to psi; the latter acts as an “extended placebo” – that is, beyond the classical conception of placebo influence, see Lucadou, 2019 – that produce unexpected fluctuations in the data. But when a “real” and robust effect is replicated by different teams of researchers, it should resist if this is not a psi effect, and this might be what happened during the reproducibility project (Nosek et al., 2015). Thus, it is still possible to demonstrate the existence of “established,” “real,” or “classical” effects, laws, or forces (which probably concern the vast majority of reality) and, in this regard, the scientific model is still totally relevant. But it means that effect sizes around 0.10 and 0.20 – the usual magnitude reported in the psi literature – in experiments within many scientific domains, might actually be the consequence of psi^20^.

The other interpretation of psi data – that psi does not exist, the “null hypothesis” (Alcock, 2003) – is also interesting from a psychological and sociological point of view. As proposed in introduction, it would suggest that hundreds of researchers (and notably more than 20 Nobel Prize winners) have been fooled for more than a century, even when using the most reliable tools of scientific research. The effect of these “illusory” results have been so convincing that they even led to practical applications (Schwartz, 2007; Mossbridge et al., 2014). For example, the United States government attempted to employ psi for more than 20 years during a program usually known as Stargate in which military personnel were selected on basis of their supposed psi-abilities to acquire information (e.g., about Russian military sites) at a distance (in space and time; Hyman, 1996; Utts, 1996; May et al., 2018)^21^. The null hypothesis would mean that staff of the best United States intelligence agencies (CIA, NSA, etc.), a number of military officers working on this program (some of whom were decorated with the legion of merit; McMoneagle and May, 2014), top scientists who have examined the project (notably a past president of the *American Statistical Association*; Utts, 1996), and even the president of the United States (Jimmy Carter admitted that a lost military plane Tu-22 has been found thanks to the Stargate program) have been fooled by the results of 504 military operations over almost 20 years (1973–1995). If this interpretation of significant results in psi experiments is accepted, it may follow that other areas of “reputable” research, involving many researchers, could also produce illusory results.

## To Conclude: Perspectives for Future Research

Psi studies are particularly interesting because whatever the reaction to the question “does psi exist?” (Bem and Honorton, 1994), their results affect the whole of psychology. If *psi does not exist*, significant results for nearly a century have only been obtained by methodological errors, self-deception, fraud, and questionable research practices. How could we avoid such a problem? Since the beginning of the replicability crisis, several solutions have been proposed – pre-registration of study designs, Bayesian statistics, larger *N*, funnel plots, *p*-curve analysis, prospective meta-analysis, adversial collaborations, etc., (Bateman et al., 2005) – which could show, at the end, non-significant results in the field of psi studies, revealing that psi was only an illusion. A pre-registration registry has already been set-up in the field of psi research^22^ (Watt and Kennedy, 2015, 2017, 2019) as well as statistical guidelines for empirical studies (Tressoldi and Utts, 2015; Kennedy, 2016; Utts and Tressoldi, 2019). Pre-submission to scientific journals which accept a paper on methodological grounds prior to results should also be promoted. In this regard, a “transparent psi project” is currently being conducted which follows these recommendations^23^. Such an approach might be extended to other psi paradigms to confirm or deny the significant results of several meta-analysis (Sherwood and Roe, 2003; Storm et al., 2010; Mossbridge et al., 2012).

On the contrary, if *psi does exist*, it means that human consciousness can interact with its environment beyond the usual boundaries of space and time, which has fundamental consequences for the way research is conducted in psychology, including psi research (as demonstrated by the psi paradox). As already mentioned, the results of experimental psi research have shown, since their beginning, strange patterns in the data (displacement, reversal, etc.) called notably psi-missing (Rhine, 1952) and elusiveness (Kennedy, 2003). A solution might be to consider these patterns not as an obstacle – or just the effect of randomness (Wiseman, 2010) – but rather as a way to better understand psi and its properties^24^. Following this idea, an original line of research has been initiated by the physicist and psychologist Walter Von Lucadou with the “Model of Pragmatic Information” (MPI; Lucadou, 1995; Lucadou et al., 2007). In this model, psi is considered as being something profoundly different to known macro-physical effects and causation, not relying on transfer of information but rather a form of entanglement process depending on the underlying nature of reality (Atmanspacher and Fuchs, 2017; Atmanspacher and Fach, 2019)^25^. A brief metaphor might be useful here. A psi experiment is like an egg where the shell forms an enclosed organizational system. It may be possible to maintain a psi effect as long as the organizational closure is not broken, that is as long as the egg is not broken to see what is inside. In this interpretation, the psi interactions are possible as long as the observer does not interfere with the system (Houtkooper, 2002). Once the system is observed, “the game is over.” This would explain why the source of psi cannot be determined precisely because the determination process would destroy the necessary conditions for the emergence of psi. It also underlines the importance of uncertainty associated with the source of psi. When the latter is used for a transfer of information, the psi effect would be suppressed, especially when attempts are made to replicate exactly the same experimental set. This is what Lucadou calls the “Non-Transmission Axiom” (Lucadou et al., 2007).

Consequently, Lucadou has tried to set-up an experiment in which this type of effect might be maintained by keeping a sufficient level of uncertainty in the system. This experiment uses the “Correlation Matrix Method” (CMM) in which the global number of correlations between the participants and an experimental task (associated with a RNG) is predicted, but not the location of such correlations in the correlational matrix (Lucadou, 2015; Flores et al., 2018; Walach et al., 2019). The non-transmission axiom could also explain the decline effect and the oscillating trends in the data (Pallikari and Boller, 1997; Maier et al., 2018; Maier and Dechamps, 2018). This last aspect is particularly interesting because these oscillating patterns might be detected, demonstrated, and analyzed when they are compared with classical effects (Rabeyron, 2014).

This line of research appears as an interesting example of what could be conceived as an example of “postmodern psychology” which takes into account the complexity of human consciousness, and more precisely postulates a potential entanglement between the observer and what is observed. It also shows how psi might be implicated in the “hard problem” of consciousness (Chalmers, 2007) or the “problem of measurement” (Wigner, 1963). Even if the possibility that psi exists sounds very implausible to many (Wiseman, 2010; Reber and Alcock, 2020), and as proposed recently by Schooler et al. (2018), a neutral and respectful approach to this topic might open heuristic debates within the wider field of psychology concerning the replicability crisis and the nature of consciousness.

## Data Availability Statement

The original contributions presented in the study are included in the article/supplementary material, further inquiries can be directed to the corresponding author.

## Author Contributions

The author confirms being the sole contributor of this work and has approved it for publication.

### Conflict of Interest

The author declares that the research was conducted in the absence of any commercial or financial relationships that could be construed as a potential conflict of interest.

## Footnotes

^1^This was the classical approach of psychical research at the beginning of the 20th century, and it has progressively disappeared in favor of studies with unselected participants (Méheust, 1999). This is problematic because the data suggest that the original method is probably more relevant (Schlitz and Honorton, 1992; May et al., 2018). The approach relying on unselected participants yields small effect sizes that can only be shown at a statistical level, and is thus easily criticized and less convincing. On the contrary, working with “gifted” participants tends to yield larger effects (Eisenbud, 1966; Braude, 2007). Three examples of this kind of experiments, conducted in control conditions, are: an historical case, with Alexis Didier who would have been able a number time to read a word and sentences in a book at a distance (Méheust, 2003); the pictures were produced by Ted Serios on a polaroid camera while he was locked in a Faraday Cage (Eisenbud, 1966); and the Pearce-Pratt experiment in which Pearce has been able to guess at a distance the right figures (among five possibilities) 25 times in a row (Rhine et al., 1966). The argument usually proposed by skeptics to explain such results is that both the experimenter and the participant were cheating (Hansel, 1961; Palmer, 2016).

^2^A meta-analysis about this effect has also reached a significant statistical effect size (*es* = 0.14; Bem et al., 2015).

^3^Psi research is not unique in this regard. It can be argued that many effects in psychology have no underlying explanation from a biological or a physical point of view. But these “classical” effects are easily accepted because they do not question the knowledge from these domains, which is the case of psi. It is indeed not usual that psychology say something about reality that could contradict sciences studying matter and living organisms.

^4^See https://psi-encyclopedia.spr.ac.uk/articles/eminent-people-interested-psi

^5^As it will be suggested later in this paper, there may be a possibility for psi researchers to avoid this path.

^6^Actually, 18% of Ganzfeld studies have been set-up without a sender and most of them still get significant results (Storm et al., 2010).

^7^Some researchers have already tried to do this with an experiment in which the participants had to mentally influence the electrodermal activity of another participant (Schlitz et al., 2006).

^8^For example, we have replicated the retro-priming experiment designed by Bem four times with a total of 344 participants (*t* = 0.92; *d* = 0.049; *CI* = −0.016, 0.16; *p* = 0.360; Rabeyron and Watt, 2010; Rabeyron, 2014; Rabeyron et al., 2018). One of the experiments has also been conducted with a population composed of artist in order to increase the effect but no significant result has been obtained (Rabeyron et al., 2018).

^9^This meta-analysis also shows that exactly replications are significant (*es* = 0.8) and that the effect mainly comes from fast thinking protocol (*es* = 0.11). The more successfully replicated protocol has been the precognitive detection of reinforcement (*es* = 0.14). For a critic of this meta-analysis, see Lakens (2015).

^10^Bem had already proposed significant results about psi since the 1990s with the Ganzfeld studies (Bem and Honorton, 1994), even after taking into account remarks from the critics.

^11^The French philosopher Méheust (1999) proposes a metaphor to describe this process close to the Sisyphus image. He describes psi research as a sweater constantly unravels. Each new generation has to “knit” again in order to maintain the existence of the sweater because new proofs are constantly necessary depending on the evolution of scientific methodology.

^12^Which could, of course, be interpreted more simply as the proof of the non-existence of psi and the “elusive argument” might be considered as a *post-hoc* hypothesis to explain non-significant results. This is why currently researchers try to demonstrate this elusive aspect of psi (Maier et al., 2018; Maier and Dechamps, 2018), but is it possible to demonstrate something that is supposed to be elusive?

^13^For example, a line of research has been developed to determine whether the nature of the targets during a psi experiment could influence the quality of the results. A significant correlation has been found between the descriptions of the participants and the degree of entropy of the target (e.g., a picture) from an informational point of view (May et al., 2000). But is it a “real” effect or the psi consequence of the belief of the experimenters (who as physicists interpret what is going on in terms familiar to them)?

^14^As explained by Cardeña (2019, p. 117–118), this problem of replicability because of statistical errors is not new and has already been described many times since the 1960s: “Just like tidal unveilings of flotsam, science discovers ‘anew’ errors of the third kind, Schlaiffer’s term for the misuse and misinterpretation of statistical procedures besides the Type I and II errors (…) Have scientists become more insightful recently about the problems of mindless applications of statistical and research procedures? No, they are just reiterating ideas that have been around for more than a half century”.

^15^The QRP hypothesis has been evaluated by Bierman (2016) employing the Ganzfeld database. He concludes that the results are probably inflated because of QRP but that they are still significant (*p* = 0.003). So the QRP hypothesis is not sufficient to explain the Ganzfeld results and, by implication, the results of many psi experiments.

^16^Classical mainstream experiments have been re-analyzed taking into account the psi hypothesis, leading sometimes to significant results. For example, Bierman (2000) has found a presentiment effect in data from classical Antonio Damasio experiments.

^17^The researchers have usually a particularly strong intention toward their results because their ability to publish in a good journal, and their own career, depends on the results they will get. Paradoxically, a scientific experiment could be considered as one of the best set-up to induce psi effect.

^18^For example, if the participant has to push a button in order to put a light in green or in red (the color of the light depends on the result of the RNG), he does not have a direct and mental influence on the RNG; he has actually choose the right time to push the button in order to select a random binary sequence (more 0 or more 1) associated with the right color.

^19^It could also be hypothesized that other controversial effects like homeopathy and different forms of complementary and alternative medicine practices (Hyland, 2003) could be the consequence of psi (Walach, 2000; Lucadou, 2019).

^20^If this hypothesis is true, it is not only important from a scientific point of view, but it also means that large amount of money invested in scientific research are lost because they actually concern psi effects. In this regard, it would be fundamental to find a way to discriminate “classical” from “psi” effects in order to avoid this problem, which can have serious consequences, especially in medical research.

^21^Such approach has also been used for searching missing persons (Schwartz, 2007), archeology (Schwartz, 2001), and financial investing (Bierman and Rabeyron, 2012).

^22^See https://koestlerunit.wordpress.com/study-registry/

^23^The results will be published in *Royal society open science*. More details here: https://psyarxiv.com/uwk7y/

^24^Another original approach consists in determining biological and genetic markers of psi. The aim is then not to demonstrate the existence of psi but rather to describe its biological foundations. For example, phenomenological and neurobiological aspects of synesthesia can be evaluated (Eagleman et al., 2007), and psi might have a lot in common with synesthesia (Simmonds-Moore et al., 2019). So, if the profound nature of psi cannot be explained, reliable markers correlated with its expression using fMRI or EEG studies could be determined in order to find neuro-correlates of psi (Moulton and Kosslyn, 2008). But the level of research in this domain has to be improved (Acunzo et al., 2013), and it should be proven that these correlations in fMRI results are not psi effects. Genetic analysis could also be relevant to detect selected participants and represent a particularly promising area of research. It is also interesting to note that a number of important discoveries have been made by working with simple organisms. A famous example has been given by a Nobel Prize winner, Kandel (2007), who has discovered molecular aspects of memory processes thanks to *Aplysia californica* – a small sea slug – equipped with a very simple nervous system (only 20,000 neurons). If psi is not a specificity of human consciousness and is shared with other species (Sheldrake, 2004), it could be relevant to look for the equivalent of *A. californica* for psi research, that is a simple biological system (plants, animals, etc.) that would allow to manipulate different variables and go beyond correlational studies.

^25^In this regard, it seems that what is observed at a macro level is close to what is described by physicists at a quantum level about the influence of the observer. The MPI relies on a mathematical formalism, the “Generalized (or Weak) Quantum Theory” (GQT; Filk and Römer, 2011; Walach and von Stillfried, 2011) which use quantum mathematical model as a metaphor. But this model does not pretend that psi is a quantum phenomenon. It rather supposes that several aspects of quantum formalism (e.g., non commutativity), which are very useful in physic, might also be relevant to describe psi processes.
